# Thermal Modeling of Tool Temperature Distribution during High Pressure Coolant Assisted Turning of Inconel 718

**DOI:** 10.3390/ma12030408

**Published:** 2019-01-28

**Authors:** Doriana M. D’Addona, Sunil J. Raykar

**Affiliations:** 1 Department of Chemical, Materials and Industrial Production Engineering, University of Naples Federico II, 80125 Naples, Italy; 2 D. Y. Patil College of Engineering and Technology, Kolhapur 416006, India; raykarsunil@gmail.com

**Keywords:** Inconel 718, turning, high pressure coolant, thermal modeling

## Abstract

This paper presents a finite-element modeling (FEM) of tool temperature distribution during high pressure coolant assisted turning of Inconel 718, which belongs to the heat resistance superalloys of the Nickel-Chromium family. Machining trials were conducted under four machining conditions: dry, conventional wet machining, high pressure coolant at 50 bar, and high pressure coolant at 80 bar. Temperature during machining plays a very important role in the overall performance of machining processes. Since in the current investigation a high pressure coolant jet was supplied in the cutting zone between tool and work material, it was a very difficult task to measure the tool temperature correctly. Thus, FEM was used as a modeling tool to predict tool temperature. The results of the modeling showed that the temperature was considerably influenced by coolant pressure: the high pressure jet was able to penetrate into the interface between tool and work material, thus providing both an efficient cooling and effective lubricating action.

## 1. Introduction

Manufacturing of aerospace components is a highly specialized industrial activity with strict materials and processing requirements. Most of the materials used for aerospace applications are exceptionally hard to machine, due to their peculiar mechanical and physical properties which are highly desirable for challenging aerospace operation environments. Several researchers are putting effort into understanding the level of machinability of aerospace alloys. These aerospace parts are expected to resist corrosion and metal fatigue without losing their strength at high temperatures and drastic temperature changes. These requirements call for the employment of high performance metal alloys for the manufacture of aerospace parts.

Nickel-based superalloys are ideal for aerospace components construction because of their high mechanical and physical properties [1]. Inconel 718 is a Heat Resistant Super Alloy (HRSA) of the Nickel-Chromium group, suitable for aerospace applications. This superalloy is widely used in the hot sections of gas turbine engines due to its superior strength and thermal behavior [2]. It is also commonly used in rocket engines, spacecraft structural components, nuclear reactors, pumps, tooling, and cryogenic tankage [3].

Earlier research has shown that Inconel 718 is one of the most difficult to cut metal alloys, as it is characterized by a high level of mechanical strength and high temperatures are generated during its machining [4]. Extreme thermal and mechanical loads close to the cutting edge during machining which lead to rapid tool wear [5]. Moreover, characteristics such as low thermal conductivity, high strain rate sensitivity favoring work hardening, presence of abrasive carbide particles, and proneness to chemically react with the tool material make its machinability furthermore critical [6,7].

In machining processes, the temperature in the cutting zone is one of the most important key issues affecting machinability, having a major impact on rapid tool wear development [8]. As temperature measurement in machining is a very difficult task, many researchers are using analytical models to obtain information regarding the temperature distribution in the cutting zone. During the machining of superalloys like Inconel 718, very high temperatures are generated due to their high thermal and mechanical properties. Accordingly, the temperature issue is a primary concern to be considered when machining this kind of superalloys because this can seriously affect the overall performance of the machining process. 

Recently, the manufacturing sector has been increasingly keen to apply sustainability at all levels of sustainability from system to products and processes. At the processes level, cutting fluids are among the most unsustainable materials and need to be addressed properly in accordance with three main and decisive aspects, also known as the triple bottom line: ecology, society, and economics. Minimum quantity lubrication (MQL) is a promising technique that minimizes the use of cutting fluids, thus improving sustainability. In Ref. [9], a review of the literature available on the use of the MQL technique during different machining processes involving titanium alloys (Ti-6Al-4V) was presented.

During metal cutting, coolant can be applied in the cutting zone using different techniques including flood cooling, Minium Quantity Lubrication, Cryogenic Cooling, and use of High Pressure Coolants. Thakur et al. [10] carried out an interesting investigation on the machining of Inconel 718 using cryogenic post-treated tungsten carbide (WC) tools. They found uniform flank wear in the case of cryogenic post treated WC tools as compared to untreated WC tools. 

Pusavec et al. [11] studied the surface integrity in cryogenic cooling machining of Inconel 718. They reported that lubricoolant fluids significantly influence the surface finish when machining with optimal parameters. When using MQL, a better surface roughness was achieved in comparison with dry machining due to the improved lubrication effect. A decrease in surface roughness was obtained by using cryogenic cooling machining versus conventional, dry and MQL machining. 

In general, adequate strategies to increase the performance of hard to cut material machining under environmental-friendly conditions have received very limited attention. The suitable selection of lubrication method and lubricants in high precision machining of difficult to cut material is of prime importance for both academic and industrial researchers.

In Refs. [12,13], a comprehensive analysis of the effect of Minimum Quantity Cooling Lubrication (MQCL) on the wear characteristic of a P25 cemented carbide cutting tool has been investigated. The scope of the research was to determine the cutting tool wear indicators and types of wear depending on the method of cutting zone cooling (dry cutting, MQCL and MQL+EP/AW, EP/AW: Extreme Pressure/Anti-Wear additives). The paper results showed that MQCL medium, as well as its correct application via generation of controllable mist can give significant improvements in cutting tool wear rate as well as productivity of cutting tool. In Ref. [14], the greatest differences in the cutting zone cooling methods MQCL and MQCL+EP/AW have been found in respect of high cutting speeds and under the highest temperature condition. Cooling under the condition of MQCL+EP/AW reduces the value of the maximum wear band by up to 27% at low cutting speeds and up to 51% at high cutting speeds as compared to the dry machining, and by 2% to 20% as compared to MQCL method. Feyzi and Safavi [15] presented the comparative study of three diverse assisted machining methods for Inconel 718, namely, plasma-enhanced machining, cryogenic cooling machining, and ultrasonic assisted machining. They found a notable improvement in surface finish with minimum surface roughness Ra value in the conventional machining method equal to 2.5 μm, whereas it was as low as 0.2 μm in the hybrid methods. Cryogenic cooling resulted in lower surface roughness of the machined parts in comparison with dry machining without noticeable increase in power consumption of the machine tool [16].

High pressure coolant (HPC) application is to date a very effective technique for coolant delivery during machining. From previous studies, suitable parameters for high pressure coolant assisted machining can be found. Diniz et al. [17] used a high pressure of 1.2 MPa (12 bar) with nozzle diameter 1.2 mm and flow rate 2.5 /min directed towards the flank face of the tool while turning AISI 1045 steel. 

Kumar et al. [18] carried out their investigation on machining ASSAB718 steel with a high pressure coolant of 17 bar. Kramar et al. [19] studied the ability of HPC in turning of surface hardened steel piston rods. The high pressure range in their investigation was 10 MPa (100 bar) with a low flow rate 0.4 /min. They also used ultra high pressure ranges between of 30–110 MPa (300–1100 bar) again with low flow rate between 0.7 and 1.4 /min. 

Palanisamy et al. [20] carried out turning of Ti6Al4V alloy with a high pressure of 90 bar. Scarce literature is available on the machining of Inconel 718 with HPC. Also, the pressure range found in previous studies ranges from 11 to 130 MPa (110 to 1300 bar), which falls into the ultra high pressure range. Nearly all of these studies were carried out with small nozzle diameters between 0.25 and 0.4 mm. Courbon et al. [21] used extremely high pressure in machining Inconel 718, such as 50, 90 and 130 MPa (500, 900 and 1300 bar) with very small nozzle diameters of 0.25, 0.3 and 0.4 mm. They found a deterioration of surface roughness at low cutting speed. They further mentioned that improvement in surface roughness can be achieved by increasing the diameter of the nozzle. With increasing diameter of the nozzle, built-up edge formation can be eliminated. 

In Refs. [22,23], the surface quality and tool behavior have been investigated during the turning of Ti-6Al-4V alloy and using coated carbide tool in dry and HPC cutting conditions. The quality of the machined surfaces was examined in respect of different cutting speed and feed rate. The tool wear was analyzed by scanning electronic microscopic (SEM) images of the worn out inserts. The improvement of surface finish, achieved as result by HPC, was attributed to the effective cooling and lubrication, low-material adhesion, reduced chip rubbing, and elimination of built-up edge.

In Ref. [24], the explorations on the machinability of Ti-6Al-4V alloy under the high-pressure coolant jets applied at the rake and flank surfaces concurrently have been studied and discussed. The machining performance has been judged with respect to cutting forces, temperature, and chip morphology defining the cooling action of HPC jets. The paper results showed that the high-pressure coolant is totally capable to produce favorable machining conditions in terms of force and temperature during turning difficult-to-cut Ti alloy.

Lopez de Lacalle et al. [25] mentioned the necessity of carrying out studies on machining Ti and Ni base superalloys at pressures in the range from 5 to 15 MPa (50 to 150 bar), which can be achieved with handy industrial equipment. For the machining of most materials, 80 bar pressure with nozzle diameter 1 mm and a flow rate of 5 /min is recommended [26]. For turning of aerospace materials, the recommended nozzle diameter is 1 mm, with a flow rate of at least 20 /min. A pressure of 70–80 bar is generally the minimum required for optimal performance [27].

Finite Element Modeling (FEM) is a very effective tool for the modeling of machining processes. As FEM techniques are known to provide accurate and precise solutions for many complex phenomena, these approaches have been applied to solve problems related to structural and thermal analysis in machining. All machining processes involve the interaction between the work piece and the cutting tool, which is a particularly complicated phenomenon. Moreover, the properties and condition of the workpiece, tool material, tool geometry, cutting parameters, machine tool dynamic performance, and clamping conditions are also important aspects that must be taken into account. This indicates that analysis of a machining process is a very difficult task, and FEM modeling can provide the correct solution to this complex problem [28]. Numerical methods using Finite Element (FE) analysis and analytical methods can predict temperatures during machining. Due to progress in analysis of numerical methods, the ability of FE models has been improved to predict machining processes, including machining forces, temperatures, residual stress, and chip morphology [29]. FE modeling is nowadays a very essential tool in current industrial practice for machining process as it can be used to model and simulate the metal cutting process before costly and time consuming experimental trials [30].

As in this investigation high pressure coolant is supplied to the cutting zone between tool and work material, it is a very difficult task to measure the tool temperature correctly. Therefore, FEM was employed as a modeling tool to predict temperature during machining of Inconel 718.

## 2. Experimental Conditions

The experimental tests were performed on a MTAB CNC turning center (MTAB, Perungudi, Chennai, India). The machine was equipped with a Kistler 9257 three-component piezoelectric dynamometer (Kistler, Milano, Italy). The coolant during the investigation was supplied with a specially manufactured high pressure coolant (HPC) system using a nozzle of 1 mm diameter. The turning tests were carried out on Inconel 718 rods (39 HRc hardness) of 28 mm diameter and 62 mm length. During the tests, the turning length was set at 28 mm. All the tests were carried out under the same machining conditions: cutting speed 100 m/min, feed rate 0.2 mm/rev, and depth of cut 1 mm. 

PVD coated carbide tools suitable for finishing and medium machining of superalloys were selected. Sandvik QM 1105 (Sandvick, Milan, Italy), TiAlN PVD coated fine-grain carbide cutting inserts with good hot hardness and toughness properties were selected. The ISO specification for this insert is CNMG 12 04 08. The tool holder was Sandvik DCLNL 2525 M12 (Sandvick, Milan, Italy) providing a rake angle of −6° and an approach angle of 95°. Turning tests were carried out under four cutting conditions: dry machining, conventional wet machining, 50 bar coolant pressure machining and 80 bar coolant pressure machining.

## 3. Thermal Modeling of Tool Temperature Distribution

**Figure 1:**
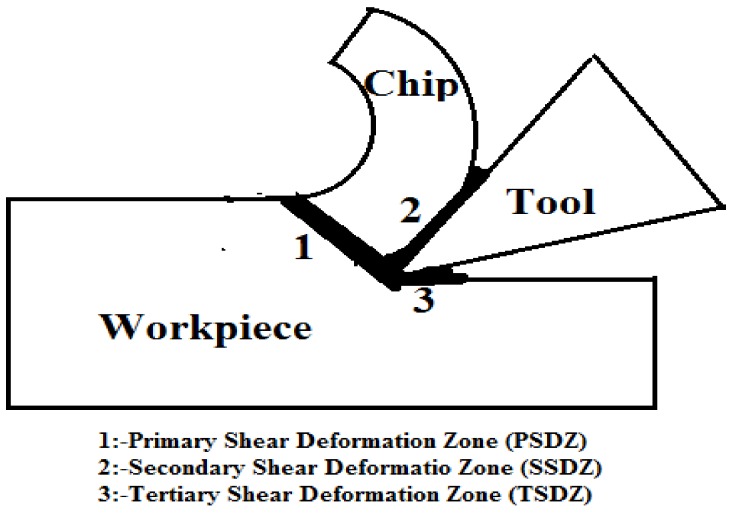
Heat generation zones during machining.

In turning operations, heat is generated in three regions during the material removal process (Figure 1). The first region is the primary deformation zone, where a large amount of heat is produced by shearing of the metal to be removed; this zone accounts for the maximum share of generated heat. The second region is the secondary deformation zone where heat is generated by the friction between the tool and the chips flowing on the tool rake face. The third region is the tertiary deformation zone, where a negligible amount of heat is generated by the rubbing of the tool flank against the machined surface. The heat generated in the primary deformation zone is mainly related to the shear force and shearing velocity. The frictional heat generated in the secondary deformation zone is also of prime importance because it can directly affect the cutting tool performance, and is related to the frictional force and the chip velocity (frictional velocity). The heat generated in the tertiary deformation zone is due to the rubbing of the tool against the machined surface and is very small in comparison to the heat of the primary and secondary deformation zones which almost account for 99% of the total heat generated during cutting [31]. Thus, in this study, only heat generation in the primary and secondary zones are considered.

The total heat generated in machining is given by Equation (1):

Total Heat Generated = Heat Generated in PSDZ + Heat Generated in SSDZ + Heat Generated in TSDZ

(1) Total Heat = Q = Qs + Qf + Qr 

By neglecting the heat generated in the tertiary deformation zone, the total heat generated is given by Equation (2):

(2) Total Heat = Q = Qs + Qf 

The heat generated in the primary deformation zone is mainly due to the plastic deformation of the work material under the action of the tool. Here, shearing takes place and chip formation initiates. This heat is essentially related to the shear force and the shearing velocity. Thus, this heat is given by Equation (3):

(3) Heat Generated in PSDZ = Qs = Fs × Vs

The shear force and the shearing velocity can be given by Equations (4) and (5) [32]:

(4) $$F_{s}=\sqrt{{\left[\left(F_{x}{\cos\;i+}_{}F_{y}\sin\;i\right)\;\cos\phi {-}_{\;}F_{z}\sin\;\phi \right]}^{2}+{\left({\;F}_{x}{\sin\;i\;-}_{}F_{y}\cos\;i\right)}^{2\;}}\;$$

(5) $$V_{s}\;=\;\left(\frac{{\cos\;\alpha }_{n\;}}{\cos\;\left(\phi {\;-\alpha }_{n}\right)}\;\right)\;\left(\frac{\cos\;i}{{\cos\;\eta }_{s}}\right)V$$

Thus, by substituting the equations of the shear force and the shearing velocity in Equation (3), the heat generated due to shearing can be given by Equation (6):

(6) $$Q_{s}=\left(\frac{{\cos\;\alpha }_{n\;}}{\cos\;\left(\phi {\;-\alpha }_{n}\right)}\;\right)\;\left(\frac{\cos\;i}{{\cos\;\eta }_{s}}\right)V\sqrt{{\left[\left(F_{x}{\cos\;i+}_{}F_{y}\sin\;i\right)\;\cos\phi {-}_{\;}F_{z}\sin\;\phi \right]}^{2}+\;{\left({\;F}_{x}{\sin\;i\;-}_{}F_{y}\cos\;i\right)}^{2}}\;$$

In the secondary deformation zone, hot chips flow against the tool rake face. Due to the velocity of the chips, a large amount of frictional heat is generated in this zone. This heat is mainly related to the frictional force and chip velocity and can be given by Equation (7):

(7) $$\mathrm{Heat}\;\mathrm{Generated}\;\mathrm{in}\;\mathrm{SSDZ}\;=\;Q_{f}=\;F_{f}\times V_{f}$$

The frictional force and the frictional velocity can be given by Equations (8) and (9) [32]:

(8) $$F_{f}=\sqrt{{\left[\left(F_{x}{\cos\;i\;+}_{}F_{y}\sin\;i\;\right){\;\sin\;\alpha }_{n}{\;-}_{}F_{z}{\;\cos\;\alpha }_{n}\right]}^{2}+{\left({\;F}_{x}{\sin\;i\;+}_{}F_{y}\;\cos\;i\right)}^{2}}$$

(9) $$V_{f}\;=\left(\frac{\mathrm{Sin}\;\phi }{\mathrm{Cos}\;\left(\phi {\;-\alpha }_{n}\right)}\right)\;\mathrm{Vc}\;$$

Thus, by substituting the equations of the frictional force and the frictional velocity in Equation (7), the heat generated due to friction can be given by Equation (10):

(10) $$Q_{f}\;=\;\left(\frac{\mathrm{Sin}\;\phi }{\mathrm{Cos}\;\left(\phi {\;-\alpha }_{n}\right)}\right)V\sqrt{{\left[\left(F_{x}{\cos\;i\;+}_{}F_{y}\sin\;i\;\right){\;\sin\;\alpha }_{n}{\;-}_{}F_{z}{\;\cos\;\alpha }_{n}\right]}^{2}+{\left({\;F}_{x}{\sin\;i\;+}_{}F_{y}\;\cos\;i\right)}^{2\;}}$$

From the equations of the heat generated by shear and friction, the total heat generated during machining can be given by Equation (11):

(11) $$Q\;=\left(\frac{\mathrm{Sin}\;\phi }{\mathrm{Cos}\;\left(\phi {\;-\alpha }_{n}\right)}\right)V\sqrt{{\left[\left(F_{x}{\cos\;i\;+}_{}F_{y}\sin\;i\;\right){\;\sin\;\alpha }_{n}{\;-}_{}F_{z}{\;\cos\;\alpha }_{n}\right]}^{2}+{\left({\;F}_{x}{\sin\;i\;+}_{}F_{y}\;\cos\;i\right)}^{2\;}}\;+\;\;\left(\frac{{\cos\;\alpha }_{n\;}}{\cos\;\left(\phi {\;-\alpha }_{n}\right)}\;\right)\;\left(\frac{\cos\;i}{{\cos\;\eta }_{s}}\right)V\;\sqrt{{\left[\left(F_{x}{\cos\;i+}_{}F_{y}\sin\;i\right){\;\cos f-}_{\;}F_{z}\sin\;\phi \right]}^{2}+{\left({\;F}_{x}{\sin\;i\;-}_{}F_{y}\cos\;i\right)}^{2\;}}\;$$

The total heat estimated by Equation (11) is taken as input to obtain the temperature distribution on the tool during steady state analysis with the ANSYS software code. Besides the heat generated, a heat transfer coefficient is also calculated by taking into consideration the high pressure coolant action. This heat transfer coefficient is also used as one of the inputs for modeling. Estimation of the heat transfer coefficient is discussed in the next section.

## 4. Tool Angle Values Employed for Analytical Modeling

The cutting tool geometry is defined by diverse angles, which play a major role in the mechanics of machining as they exert a major influence on surface roughness, cutting forces, tool wear and heat generated in machining. The following tool angles are considered in this investigation for modeling [32,33].

(12) $${\mathrm{Normal}\;\mathrm{Rake}\;\mathrm{Angle}\;=\;\alpha }_{n}{=\;\tan}^{-1}\left(\tan\alpha \;\cos i\right)$$

(13) $$\mathrm{ShearAngle}=\phi \;{=\;\tan}^{-1}\left(\frac{r_{c}\cos\;\alpha_{n}}{{1-r}_{c}\sin\;\alpha_{n}}\right)$$

(14) $${\mathrm{Shear}\;\mathrm{Flow}\;\mathrm{Angle}\;=\;\eta }_{s}{=\;\tan}^{-1}\left\{\frac{\left[\tan\;i\;\cos\;\left(\phi -\;\alpha_{n}\right)\;-\;\tan\;\eta_{c}\;\sin\;\phi \;\right]}{\cos\;\alpha_{n}}\right\}$$

(15) $${\mathrm{Chip}\;\mathrm{Flow}\;\mathrm{Angle}\;=\;\eta }_{c}{=\;\tan}^{-1}\left\{\frac{\;\sin\;\left(k_{r.\mathrm{avg}}{+\;C}_{b}\right)}{\;\left[2d/\left({f\;\sin\;k}_{r.\mathrm{avg}}\right)\;+\;\cos\;\left(k_{r.\mathrm{avg}}{\;+\;C}_{b}\right)\right]}\right\}$$

where k_r.avg_ can be given as

(16) $$k_{r.\mathrm{avg}}=\frac{\left\{\left[\;\left(d/r\right){\;+\;\cos\;k}_{r}–\;1\right]\;\left({1/\sin\;k}_{r}\right)\;+\;\left(k_{r}/2\right)\right\}}{\left[\;\left(d/r\right){\;+\;\cos\;k}_{r}–\;1\right]/\left(k_{r}\;{\sin\;k}_{r}\right)\;+\;1}$$

## 5. Effect of the High Pressure Coolant

**Figure 2:**
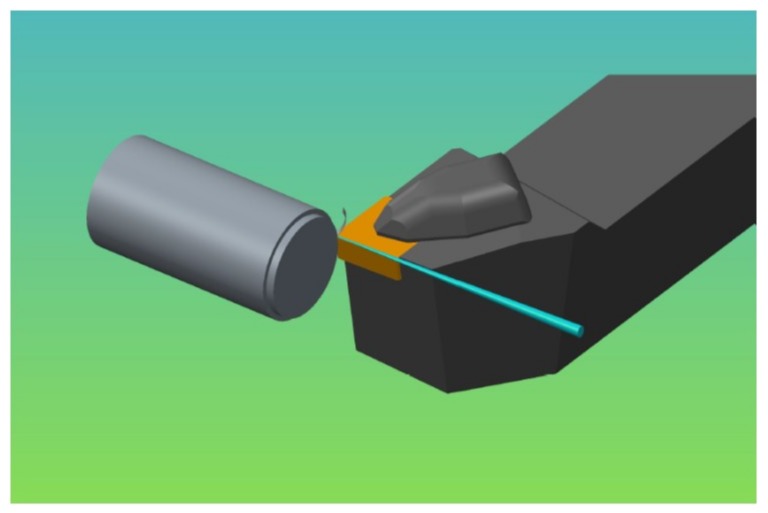
Direction and location of high pressure coolant application.

In this investigation, a pressure coolant jet was supplied to the machining zone. The coolant was applied in such a way that it was directed exactly at the tool tip where the tool is in contact with the workpiece and where the chip originates, as shown in Figure 2. In this manner, it provided the combined benefits of both kinds of coolant application: from the tool flank side and from the tool rake side. A high pressure coolant is able to remove the heat from the machining zone at a very high rate due to the high velocity of the pressurized coolant. As the coolant velocity is very high, the flow of the fluid is considered to be turbulent. To see the effect of the turbulent flow of the coolant, the convection heat transfer coefficient was calculated for different coolant pressure values. The heat transfer coefficient can be expressed in terms of Nusselt number [34] by Equation (17):

(17) $$\mathrm{hc}\;=\;\frac{\mathrm{Nu}\left(x\right)\;\times \;\lambda }{x}$$

The Nusselt number for a forced convection and turbulent flow is a function of the Reynolds and Prandtl dimensionless numbers, as given by Equation (18):

(18) $$\mathrm{Nu}\;=\;0.029{\mathrm{Pr}}^{1/3}\;\times \;R{\left(x\right)}^{4/5}$$

The Nusselt number for a convective heat transfer can also be expressed in terms of heat transfer coefficient and distance characteristic of the fluid, as given by Equation (19). This distance characteristic in turning is the distance from which the fluid is applied to the cutting zone.

(19) Nu(x) = hc × xλhc=Nu (x) × λxhc=0.029 Pr1/3 × Re (x)4/5 × λx

The Reynolds number is a dimensionless quantity, which is very useful when deciding the nature of the flow, i.e., whether it is lamellar or turbulent. It is related to distance characteristics, viscosity, density, and velocity of the fluid coming out of the nozzle, as given by Equation (20):

(20) Re(x) = ν × xμ × ρ 

The Prandtl number is also a dimensionless quantity, which depends on the specific heat and viscosity of the fluid, as given by Equation (21):

(21) $$\mathrm{Pr}\;=\;\frac{\mathrm{Cp}}{\lambda }\;\times \;\mu \;$$

The velocity of the fluid can be expressed by Equation (22):

(22) Vcoolant = Fq/An

**Figure 3:**
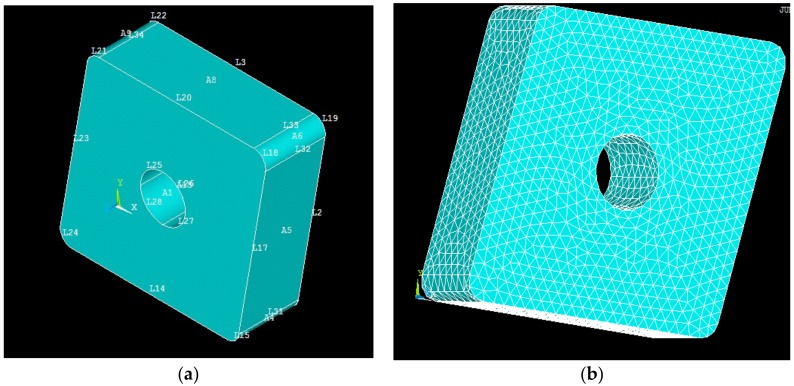
(**a**) Insert model (ANSYS); (**b**) Insert model with a fine mesh (ANSYS).

The temperature during any machining operation is a very critical quantity to measure. In high pressure coolant assisted turning operations, a high pressure and velocity fluid flows into the cutting zone. In practice, it is very difficult to measure temperature during turning, and this becomes even more impractical when machining alloys like Inconel 718 with high pressure coolant. Therefore, the actual temperature was not experimentally measured in this study. Table 1 reports thermal and mechanical properties of the work material and the tool material used during the FEM analysis. These properties are provided by tool and workpiece material manufacturers. In current investigation (Figure 3), a steady state thermal analysis is carried out in ANSYS 14. For this aim, a 3D model is drawn using NX-4 CAD module and then imported into ANSYS. For the steady state thermal analysis, the body of tool is considered as Tetrahedron element. Once the meshing is done, boundary conditions mentioned below are applied on insert body. Heat generated, calculated using analytical models, are applied at the nose of the tool. After the processing, a solution for temperature is obtained.

When a high-pressure coolant is introduced into the cutting zone, the jet will remove heat from the interface and carry it away from the machining zone. In this investigation, a cooling effect by the jet is described by using a convective heat transfer coefficient, described in Equations (17)–(19). For every finite element model, boundary conditions are very important to get accurate results. The following boundary conditions are used during this analysis:

- The internal surfaces of the insert, which are in contact with the shim seat and the holder, are assumed to be smooth and in perfect contact.
- For the exterior, boundaries of the insert which are exposed to the air a heat transfer coefficient of h = 20 W/m^2^ is considered.
- Initially, the whole model is kept at an ambient temperature of 20 °C.

## 6. Results and Discussion

**Figure 4:**
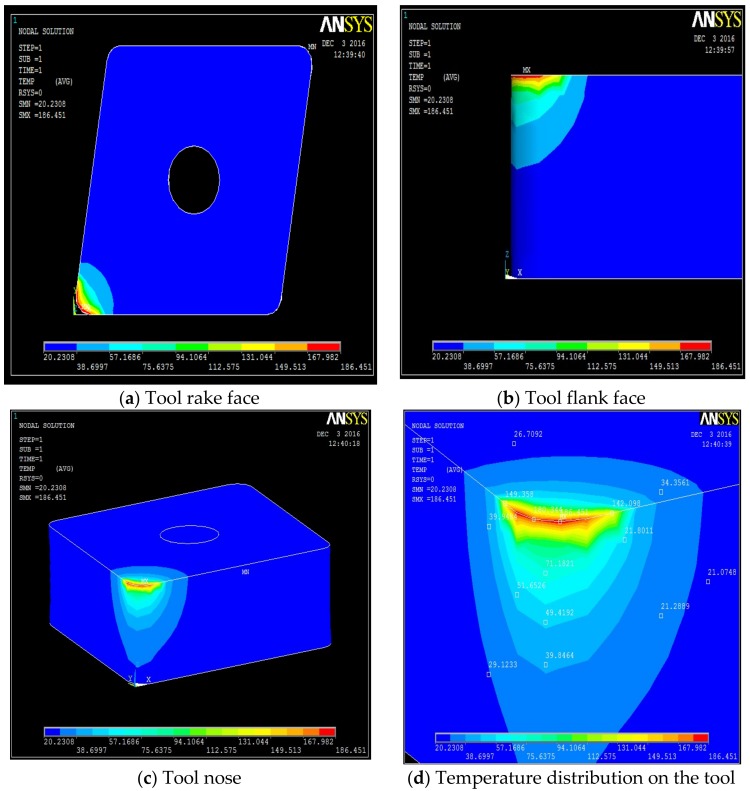
Dry machining temperature fields on the tool, along its different faces. (**a**) Tool rake face, (**b**) tool flank face, (**c**) tool nose, (**d**) temperature distribution on the tool.

The temperature distribution can be observed on different faces of the tool. Table A1, Table A2, Table A3 and Table A4 show the data calculated using the analytical models defined by Equations (1)–(21). It is found from the results of the FEM analysis that the temperature at the tool nose for different machining conditions are 186.45 °C for dry machining, 179.15 °C for conventional wet machining, 151.03 °C for 50 bar pressure coolant and 147.06 °C for 80 bar pressure coolant machining. These temperature zones are observed at the nose of the tool tip. This is observed in FEM models as well as on actual tool tip images shown in the Figure 4.

Steady state analysis with the ANSYS code indicates that a temperature drop of 38 °C and 31 °C is observed when machining with 80 bar coolant pressure as compared to machining with dry and conventional wet machining, respectively. When machining at 50 bar coolant pressure, a drop of 35 °C and 28 °C, is observed, as compared to dry and conventional wet machining, respectively.

**Figure 5:**
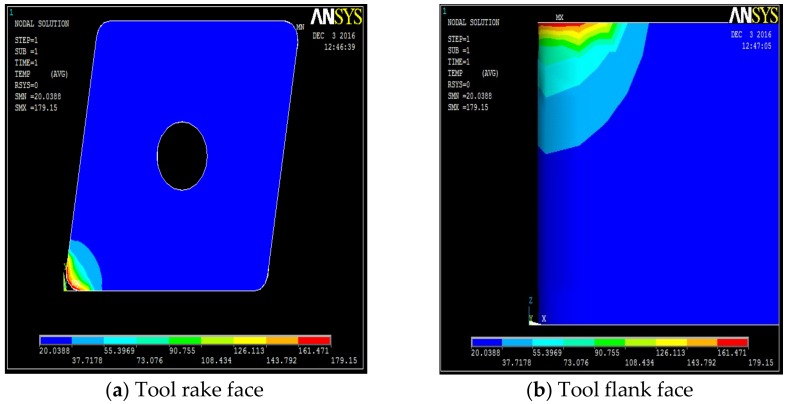

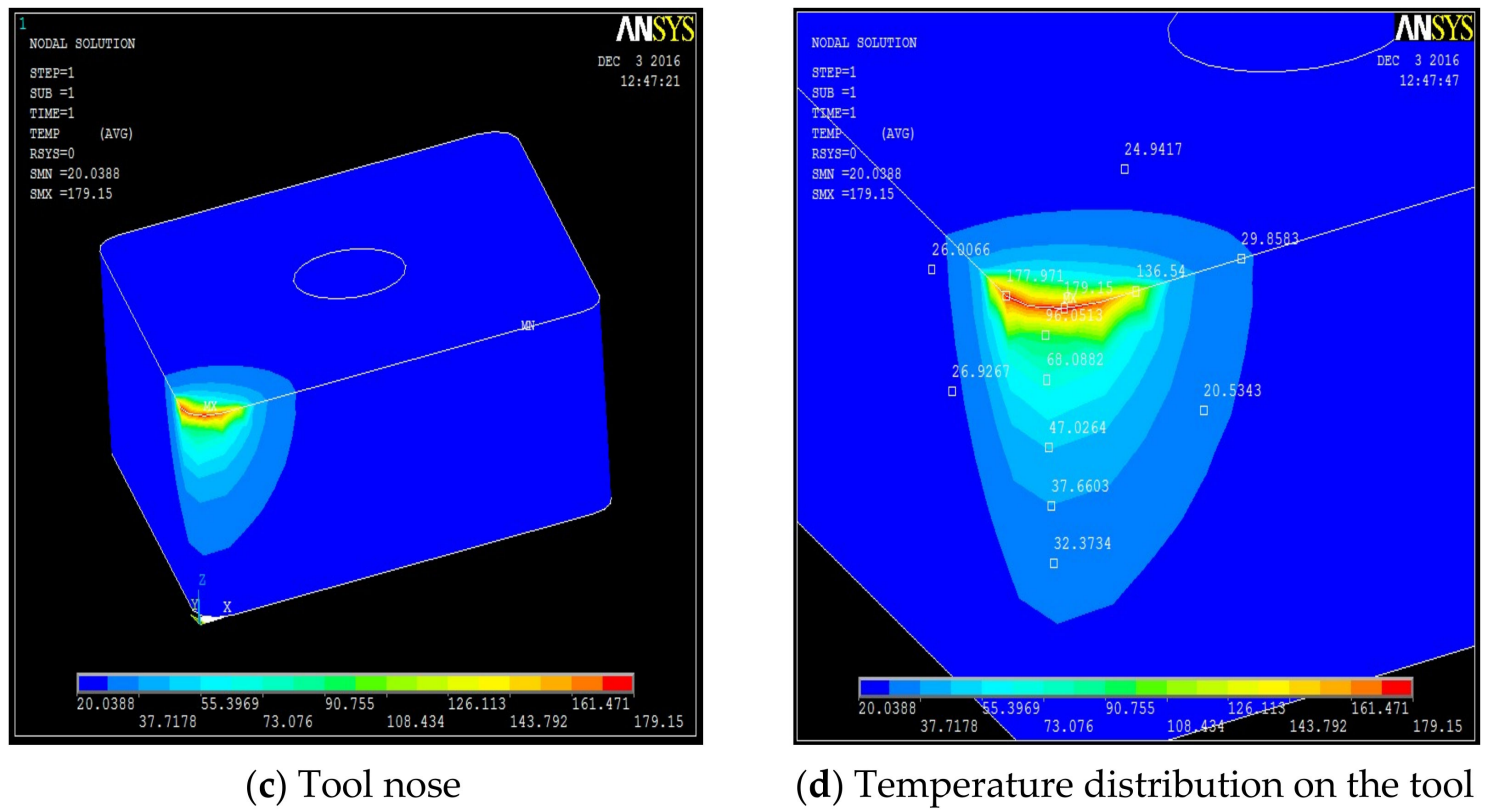
Wet machining temperature fields on the tool, along its different faces. (**a**) Tool rake face, (**b**) tool flank face, (**c**) tool nose, (**d**) temperature distribution on the tool.

**Figure 6:**
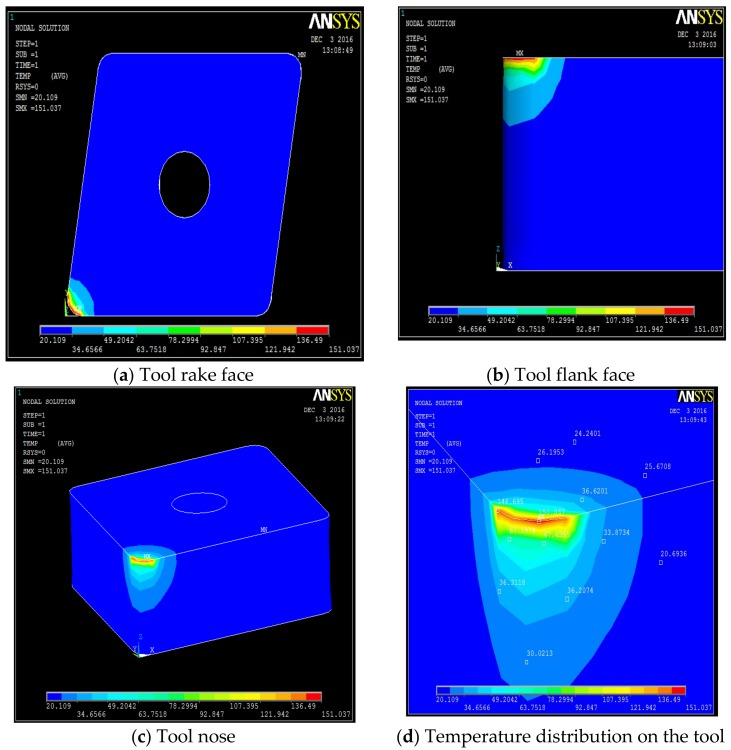
Machining with 50 bar: temperature fields on the tool, along its different faces. (**a**) Tool rake face, (**b**) tool flank face, (**c**) tool nose, (**d**) temperature distribution on the tool.

**Figure 7:**
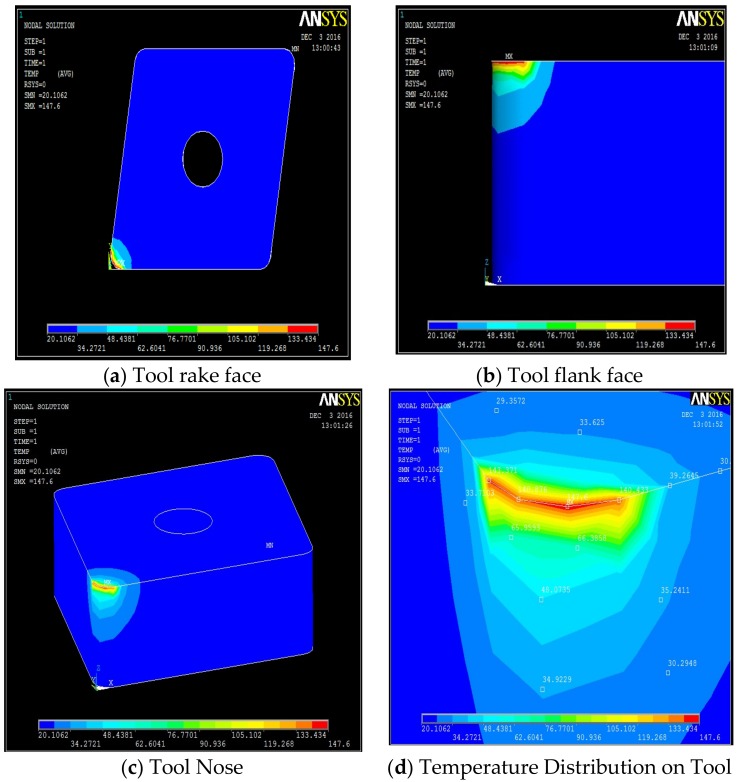
Machining with 80 bar: temperature fields on the tool, along its different faces. (**a**) Tool rake face, (**b**) tool flank face, (**c**) tool nose, (**d**) temperature distribution on the tool.

A high pressure jet is able to partially penetrate into the cutting interface, thus providing an efficient cooling and effective lubricating action. The coolant liquid wedge created at the tool–tip interface reduces the tool–chip contact length, lowers the coefficient of friction and, consequently, decreases the temperature during machining. The above trend is because of the reduced frictional conditions at the tool-chip interface. This condition can be attributed to the formation of chips during the machining. An increase in pressure also increases velocity of the coolant and exerts higher impact on the chips, which breaks the chips into small pieces. From the flow rate and cross sectional area of the nozzle, the velocity of coolant at different pressure conditions can be calculated as V_coolant_ = Fq/An. Velocity of the coolant at different pressures were 52.22 m/s, 84.84 m/s, and 127.38 m/s at 20 bar, 50 bar and at 80 bar respectively. In addition, a rise in pressure increases force exerted by coolant jet on the chip. Force exerted by a jet is equal to ρA_n_V_coolant_^2^ [35] where ρ is density of coolant. Therefore, a force of 2.14 N at 20 bar, 5.64 N at 50 bar, and 12.72 N at 80 bar is exerted on the chip due to pressure of the coolant. This impact exerted due to high velocity of the coolant at high pressure promotes curling of chips at a faster rate than at low pressure due to its high velocity and force. Temperature views, temperature distribution on different faces of the tools, and temperature variation with respect to time for dry machining, wet machining, 50 bar coolant pressure, and 80 bar coolant pressure machining are shown in Figure 4, Figure 5, Figure 6 and Figure 7.

**Figure 8:**
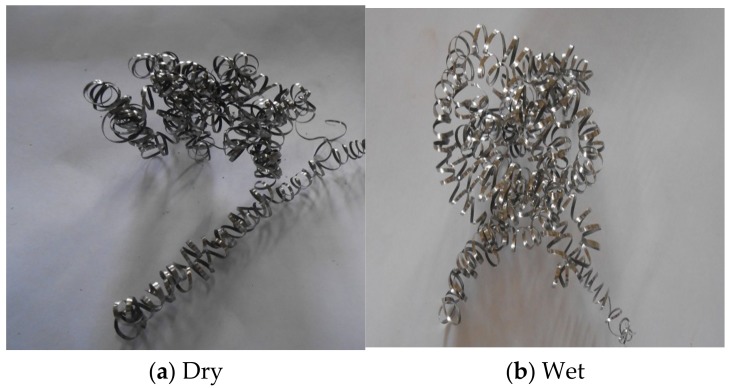
Chip Forms at different machining conditions. (**a**) Continuous chip during dry cutting; (**b**) continuous chip during wet cutting.

**Figure 9:**
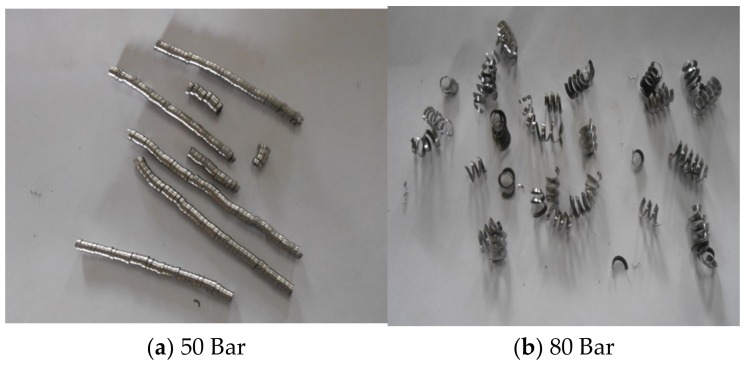
Chips Forms at Various Machining Conditions. (**a**) Long chip form; (**b**) short chip form.

**Figure 10:**
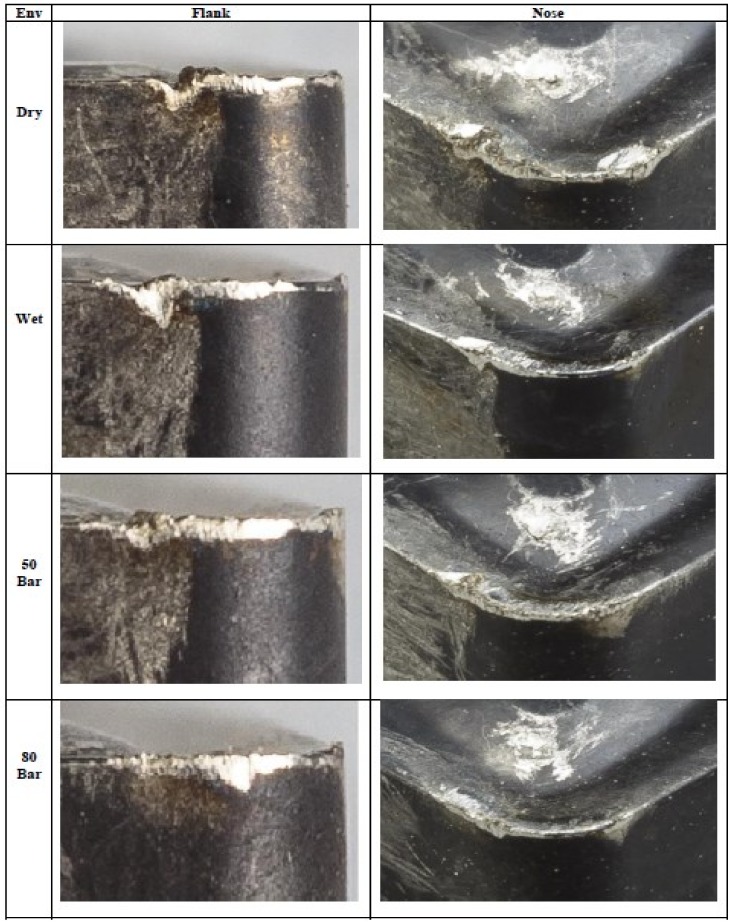
Tool Wear Patterns at Different Machining Conditions.

High pressure improves curling, forming spring type chips as shown in Figure 8. When coolant is applied at high pressure, it breaks the chips into small pieces and keeps them away from the cutting zone. This decreases the length of contact of chips with the tool. The improved breakability and the curling action of chips results in less friction at the tool-chip interface. It is observed from the analysis that during dry machining, the tool gets worn out immediately (Figure 9). This is because during dry machining, a large amount of heat is generated during turning of Inconel 718. This is visible during the machining also as in the cutting region continuous sparks are generated during actual machining. Therefore, the tool gets softened very quickly during the machining, which causes rapid wear of the tool. Built up edge formation and burn marks are also visible on the tool during dry machining. These patterns are visible in Figure 10.

## 7. Conclusions

Finite Element Modeling (FEM) was carried out to obtain the temperature distribution on the tool and the effect of coolant pressure in reducing the tool temperature during turning of Inconel 718. Coolant pressure was considered the most important parameter in formulating the model. For this purpose, a heat transfer coefficient based on Nusselt, Reynolds, and Prandtl numbers was calculated. FEM models confirmed that the temperature is significantly influenced by the pressure of the coolant (Table A4). The high pressure jet was able to partially penetrate into the cutting interface, thus providing both an efficient cooling and lubricating action. The coolant liquid wedge created at the tool–chip interface reduced the tool–chip contact length, decreased the coefficient of friction and, consequently, lowered the temperature during machining. Steady state analysis with the ANSYS code indicated that a temperature drop of 38 °C and 31 °C was observed when machining with 80 bar coolant pressure as compared to machining with dry and conventional wet machining, respectively. When machining at 50 bar coolant pressure, a drop of 35 °C and 28 °C was observed, as compared to dry and conventional wet machining, respectively. Though actual temperature was not measured during this investigation, the heat partitions were calculated from cutting force components which were measured with a dynamometer.

Future research will entail deeper studies of high pressure coolant assisted turning of Inconel 718 investigating new pressure coolant ranges and using the FEM as a modeling method to predict the tool temperature distribution in order to expand the effectiveness and reliability of the proposed approach.

## Figures and Tables

**Table 1:** Thermal and mechanical properties of the work material and the tool material.

ID	Property	Work Material	Tool Material
1	Poission’s ratio	0.29	0.22
2	Young’s modulus	205 GPa	534 GPa
3	Thermal conductivity	11.4 W/m/°C	50 W/m/°C
4	Density	8200 kg/m^3^	11900 kg/m^3^
5	Specific heat	435 J/kg/°C	400 J/kg/°C

## Nomenclature

F_c_	Main cutting force	(N)
F_p_	Radial/thrust force	(N)
F_f_	Feed force	(N)
F_s_	Shear Force	(N)
F_fr_	Frictional force	(N)
F_n_	Normal force	(N)
α	Rake angle	(°)
αn	Normal rake angle	(°)
i	Inclination angle	(°)
ηs	Shear flow angle	(°)
ηc	Chip flow angle	(°)
rc	Chip thickness ratio	
φ	Shear angle	(°)
Kr	Approach angle	(°)
r	Tool nose radius	(mm)
C_b_	End cutting edge angle	(°)
ρ	Density of work material	(kg/m^3^)
μ	Coefficient of friction	
Vs	Shear velocity	(m/s)
Vf	Frictional Velocity (Chip velocity)	(m/s)
Qf	Frictional heat	(N·m/s)
Qs	Heat at shear zone	(N·m/s)
f	Feed rate	(mm/rev)
Vv	Cutting Speed	(m/min)
a_p_	Depth of cut	(mm)
Nu	Nusselt number	
Pr	Prandtl number	
Re	Reynolds number	
x	Distance characteristics	
Te	Effective Tool Temperature	(°C)
Co	Kronenberg’s constant	
K_s_	Unit force	(N/mm^2^)
A_c_	Chip cross-sectional area	(mm^2^)
W	Specific heat	
H	Thermal conductivity of material	(W/m/°C)
V_coolant_	Velocity of Coolant	(m/s)
PSDZ	Primary Shear Deformation Zone	
SSDZ	Secondary Shear Deformation Zone	
TSDZ	Tertiary Shear Deformation Zone	
V_coolant_	Velocity of Coolant	(m/s)
F_q_	Flow rate of coolant	
λ	Thermal Conductivity of fluid (coolant)	(W/m/°C)
ν	Viscosity of Fluid (coolant)	
Cp	Specific Heat of Fluid (coolant)	
A_n_	Cross-sectional area of nozzle	
MQL	Minimum Quantity Lubrication	

## Appendix A

**Table A1:** Calculation of the shear angle for different conditions.

ID	Environment	V_c_	f	ap	CT	r_c_	cosα_n_	sinα_n_	Ø
1	DRY	100	0.2	1	0.336	0.595	0.372	0.995	29.14
2	WET	100	0.2	1	0.322	0.621	0.413	0.995	30.12
3	50 bar	100	0.2	1	0.319	0.627	0.453	0.995	30.34
4	80 bar	100	0.2	1	0.308	0.649	0.494	0.995	31.17

**Table A2:** Calculation of the friction and the shear force for different conditions.

CON	F_c_	F_p_	F_f_	Cos i	Sin i	Sin α_n_	Ø	Sin Ø	Cos Ø	F_fr_	F_s_
**DRY**	651.5	294.4	312.1	0.995	−0.105	−0.104	29.140	0.55	0.83	436.96	361.38
**WET**	615.9	275.5	299.6	0.995	−0.105	−0.104	30.120	0.56	0.83	413.34	338.86
**50 bar**	572.7	274	275.9	0.995	−0.105	−0.104	30.340	0.57	0.82	397.62	332.82
**80 bar**	502.7	272.4	224.7	0.995	−0.105	−0.104	31.170	0.58	0.82	367.55	323.87

**Table A3:** Calculation of velocities and heat for different conditions.

EN	Ø	Cos (Ø-α)	ηs	Vf	Vs	F_fr_	F_s_	Qf	Qs	Total
**DRY**	29.14	0.82	−6.49	0.992	2.027	436.96	361.38	433.27	732.65	1165.92
**WET**	30.12	0.81	−6.48	1.034	2.052	413.34	338.86	427.57	695.42	1122.99
**50 bar**	30.34	0.81	−6.47	1.044	2.058	397.62	332.82	415.20	684.93	1100.13
**80 bar**	31.17	0.80	−6.46	1.082	2.080	367.55	323.87	397.53	673.75	1071.28

**Table A4:** Heat transfer coefficient for different pressure conditions.

Pressure	Reynolds Number (Re)	Prandtl Number (Pr)	Convection Heat Transfer (hc)
20 bar	190833.90	1289.88	19997.008
50 bar	305363.1	129.88	29097.740
80 bar	458044.52	129.8	40246.887
